# Effects of auditory feedback on fine motor output and corticomuscular coherence during a unilateral finger pinch task

**DOI:** 10.3389/fnins.2022.896933

**Published:** 2022-11-03

**Authors:** Jing Guo, Tian Liu, Jue Wang

**Affiliations:** ^1^School of Life Science and Technology, The Key Laboratory of Biomedical Information Engineering of Ministry of Education, Institute of Health and Rehabilitation Science, Xi’an Jiaotong University, Xi’an, Shaanxi, China; ^2^National Engineering Research Center for Healthcare Devices, Guangzhou, China; ^3^The Key Laboratory of Neuro-informatics and Rehabilitation Engineering of Ministry of Civil Affairs, Xi’an, Shaanxi, China

**Keywords:** precise motor control, auditory feedback, power spectrum, beta band, corticomuscular coherence

## Abstract

Auditory feedback is important to reduce movement error and improve motor performance during a precise motor task. Accurate motion guided by auditory feedback may rely on the neural muscle transmission pathway between the sensorimotor area and the effective muscle. However, it remains unclear how neural activities and sensorimotor loops play a role in enhancing performance. The present study uses an auditory feedback system by simultaneously recording electroencephalogram (EEG), electromyography (EMG), and exert force information to measure corticomuscular coherence (CMC), neural activity, and motor performance during precise unilateral right-hand pinch by using the thumb and the index finger with and without auditory feedback. This study confirms three results. First, compared with no auditory feedback, auditory feedback decreases movement errors. Second, compared with no auditory feedback, auditory feedback decreased the power spectrum in the beta band in the bimanual sensorimotor cortex and the alpha band in the ipsilateral sensorimotor cortex. Finally, CMC was computed between effector muscle of right hand and contralateral sensorimotor cortex. Analyses reveals that the CMC of beta band significantly decreases in auditory feedback condition compared with no auditory feedback condition. The results indicate that auditory feedback decreases the power spectral in the alpha and beta bands and decreases corticospinal connection in the beta band during precise hand control. This study provides a new perspective on the effect of auditory feedback on behavior and brain activity and offers a new idea for designing more suitable and effective rehabilitation and training strategies to improve fine motor performance.

## Introduction

Auditory feedback is a way to utilize auditory feedback messages to assist participants in coordinated motion control, and regulating kinematic parameters in sundry devices, including the fields of sports and rehabilitation (Sigrist et al., 2013). In terms of rehabilitation, the influence of auditory feedback may increase the fine motor ability of patients with writer’s cramps (Baur et al., 2009). Furthermore, it could improve fine sports skills such as precision shooting (Konttinen et al., 2004). Besides, auditory feedback can be used in various precise motor tasks, such as handwriting (Danna et al., 2015), balancing tasks (Rath and Rocchesso, 2005), and finger tasks (Hwang et al., 2018). Studies used concurrent auditory feedback through the sonification of performance that maps the movement data into an auditory message (such as rhythm, loudness, and pitch), this process can improve motor control by adjusting motor output (Schaffert et al., 2019). Although numerous studies show that auditory feedback associated with tasks results in improved precise motor performance, the role of cortical synchronous oscillates with muscles in this process remains unclear.

To elucidate the corresponding mechanism related to auditory feedback to improve the movement output, this research employed corticomuscular coherence (CMC) to serve as a pivotal means. CMC is a necessary neurophysiological approach to capture the synchronous connection between the contralateral sensorimotor region and the corresponding muscle in motor control (Mima and Hallett, 1999). Previous studies investigated that CMC of the beta band was obvious during weak (Kristeva et al., 2007; Johnson et al., 2011), moderate (Hashimoto et al., 2010), and constant muscle contraction and vanished during movement (Mehrkanoon et al., 2014). Besides, CMC of the beta band can be transmitted through the corticospinal tract, these studies recommended that CMC of the beta band is associated with the maintenance of sensorimotor status (Liu et al., 2019). The current knowledge from only a few studies indicated that sensorimotor feedback can alter the CMC of the beta band. Despite these studies using CMC in sensorimotor integration such as visuomotor task (L’Abbate et al., 2022) and tactile feedback task (Li et al., 2020), no study has been carried out on the corticospinal pathway of auditory feedback task in our knowledge. As is known to all, auditory feedback is important information for sensory feedback (Rath and Rocchesso, 2005; Park et al., 2015; Boyer et al., 2017; Gao et al., 2018). Auditory feedback could modulate motor performance. This process involves complex neural control mechanism (Schmitz et al., 2013; Effenberg et al., 2017). Audio-motor coupling of cortex level holds the view that auditory network and motor network activate coordinately. In cortex-level network analysis with fMRI data is the suitable tool to analyze the audio-motor coupling mechanism due to high spatial resolution (Schmitz et al., 2013). CMC analysis relies on the “internal model” theory (Kawato, 1999; Wolpert and Ghahramani, 2000). Auditory feedback is based on the audio-motor coupling hypothesis. This hypothesis holds the idea that audio-motor constructs feedforward pathways and feedback pathways to perform precise movement (Zatorre et al., 2007). Auditory feedback regulates motor performance by mapping sound and movement output. Feedforward information predicts motor through “efference copy,” feedback information transfers auditory information to the sensorimotor cortex, compares the target sound to the received sound through the internal model, and then modifies the movement (Buxbaum et al., 2005; Rodriguez-Fornells et al., 2012). Meanwhile, precise motor control generally requires corticospinal pathway at the anatomical level (Lemon and Mantel, 1989). Previous studies shown that corticospinal pathway could measure through CMC (Conway et al., 1995). CMC computes the synchronous coupling between the contralateral sensorimotor region and the corresponding muscle, it can represent different status of sensorimotor (Mima and Hallett, 1999). Hence, the purpose of the current study is to investigate how does the auditory feedback influent the CMC between nerve and muscle in auditory motor task. Here, we hypothesize that the beta band CMC will be altered in the somatosensory cortex during different auditory feedback conditions.

To test this hypothesis, we manipulate the electroencephalogram (EEG), electromyography (EMG), and force signal to study the effect of auditory feedback during precise thumb and index finger pinching tasks. The present study uses EEG and EMG coherence of the corticomuscular pathway to assess how the presence and absence of auditory feedback influence the processing of sensorimotor information. The present research may provide a new viewpoint on the corticomuscular interaction with and without auditory feedback and promote the comprehension of potential cortico-muscular pathways on how the brain and muscle system optimize the control output strategy during precise movement.

## Materials and methods

### Participants

Nineteen healthy volunteers were recruited in this experiment in total. The force output of the two participants did not meet our behavior criterion (The exerted force during the steady-hold period was outside the 2–6 N force range). More than half of the trials were removed, thus, the present study excludes their data in the following analysis. Two participants performed badly, which may be due to the following reasons: participants’ maladjustment to the auditory feedback task, participants’ distractibility, individual differences in motor control, strict selection criteria of trial, and experimental process control. Eventually, 17 healthy volunteers, between 22 and 32 years of age (mean age 25.8 years, eight men and nine women) were included. Every participant was self-proclaimed to be a right-hander before the experiment. Participants had normal hearing and no disorders related to the nervous system or the musculoskeletal system. All of them came from Xi’an Jiaotong University and gave informed consent to the experimental procedure. The procedures and protocol were in accordance with the Helsinki declaration and approved by the Ethics Committee of the Medical College of Xi’an Jiaotong University.

### Experimental design and task

**FIGURE 1:**
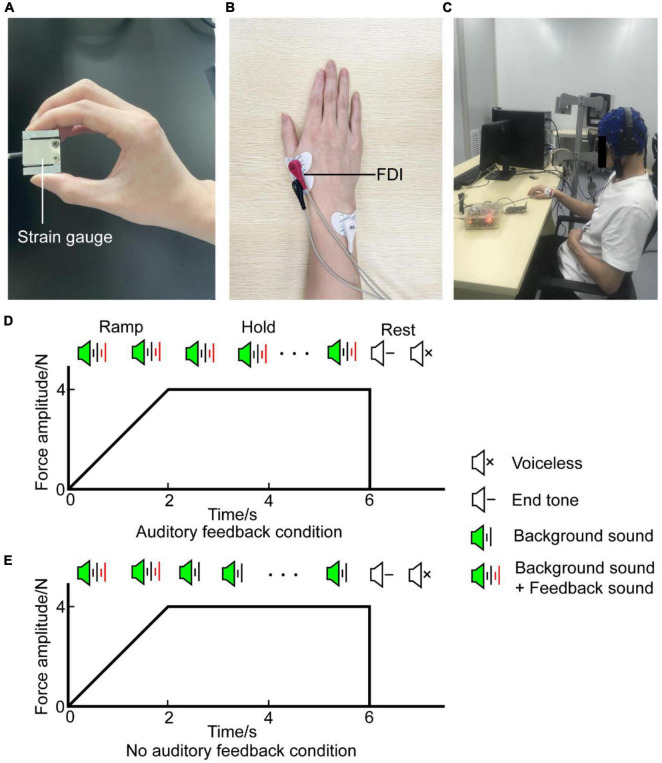
Experiment setup. **(A)** Precision grip strain gauge held between the thumb and index finger by the participant during the task. **(B)** EMG signal was obtained from the rFDI muscle. **(C)** The participant was seated in an armchair with an EEG cap. **(D)** Diagram of auditory feedback condition. A task with auditory feedback during the steady-hold period. **(E)** Diagram of no auditory feedback condition. A task without auditory feedback during the steady-hold period. In **(D,E)**, the horizontal axis means the time, the vertical axis means the force amplitude. Each trial was composed of a ramp period and a steady-hold period. The ramp period lasted from 0 to 2 s, the steady-hold period lasted from 2 to 6 s. After a rest period of about 1.5 s, participants processed the next trial. EMG, electromyography; rFDI, right first dorsal interosseous.

Participants receive the auditory signals by headphones (MDR-ZX310, Sony, Japan) binaurally and are instructed to generate a target force of about 4N (target interval: 3.6N∼4.4N) by gripping the force sensor between their right thumb and index finger (Figure 1A). EMG activity was recorded with the bipolar channel of the SynAmps^2^ system (NeuroScan Inc., USA) by adhesive surface electrodes from the right first dorsal interosseous (rFDI) muscle (Figure 1B). Participants were comfortably seated in a chair located in a dim and quiet room, their left forearm sagged naturally and their right forearm was placed on the armrest beside them (Figure 1C). Each participant confirmed that they can perceive the auditory signals before the experiment. Trials involve two voices: feedback sound and background sound. Background sound is a cosine-modified signal of 1,000 Hz lasting 50 ms, its rising edge and falling edge are 10 ms, respectively. Its volume stays constant. Feedback sound is divided into white noise and pink noise according to the magnitude of the output force. Noise volume is proportional to the difference between the target force and exerted force. When the produced force is higher than the maximal value of the target interval force, the sound is white noise combined with background sound, when the produced force is lower than the minimal value of the target force, the sound is pink noise combined with background sound, when the produced force within the target interval force, the sound is background sound. The sounds were created with written MATLAB scripts in the psychology toolbox (Brainard, 1997).

Every trial lasts for 6 s. In the first 2 s, participants are instructed to produce target force as soon as possible when the sound is onset. This phase is named the ramp period. Then maintain target force in the last 4 s with or without auditory feedback, this phase is named the steady-hold period. After the steady-hold period, an end tone reminds the participants to rest for about 1.5 s, then, the participants would process the next trial (Figures 1D,E). Therefore, the task involves two conditions: auditory feedback and no auditory feedback. In the auditory feedback condition, the sounds include feedback sound and background sound during the ramp period and steady-hold period. In the no auditory feedback condition, the sounds include feedback sound and background sound during the ramp period, and sound includes background sound during the steady-hold period. First, participants performed serval pre-experiment blocks to familiarize themselves with the procedure. Each pre-experiment block contains 10 trials. Then, each participant completes four task blocks, each task block contains 40 trials, 20 trials had auditory feedback and the remaining half had no auditory feedback in the steady-hold period. Two conditions are randomly distributed in each block. Besides, several minutes of rest is needed to avoid fatigue between every block interval. In total, the experiment protocol lasts for 30 min approximately.

### Data acquisition

Participants wore a collecting cap with 30 Ag/AgCl EEG electrodes according to the extended 10–20 system installed on it. Surface EEG was acquired using a SynAmps^2^ system (NeuroScan Inc., USA). A ground electrode was placed on the forehead, the reference electrode was placed on the right mastoid. Impedances of all electrodes were kept below 10 kΩ. The continuous EEG and EMG were digitized with a sampling rate of 1,000 Hz and band pass filter from 0.05 to 400 Hz. A strain gauge (JLBS-M2, s-type tension-compression sensor, Bengbu Sensor System Engineering Co., Ltd., China, 30 mm × 25 mm × 13.5 mm, full scale: 10N, accuracy: 0.05% full scale) was applied to record the exerted force by the participants. The force digitized from the analog force data at 200 Hz.

### Data processing

**FIGURE 2:**
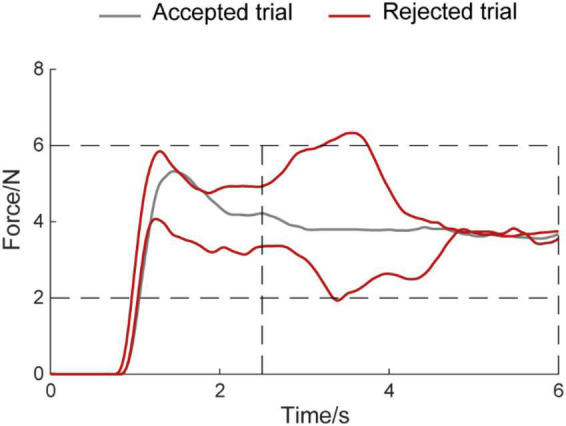
Example diagram for accepted trials and rejected trials. The horizontal axis means the time, the vertical axis means the force amplitude. The Gray line means accepted trial, the red line means rejected trial.

Entire analyses were carried out using MATLAB (MATLAB R2016a, The MathWorks Inc.) including power spectrum, CMC, and statistical analysis. Besides, the computation of power spectrum and CMC was performed in FieldTrip, an open-source MATLAB toolkit for neurophysiological data analysis (Oostenveld et al., 2011). Before the calculation of the power spectrum and CMC, the following processing steps were conducted. First, the raw EEG was browsed visually to identify whether the data contains bad channel person by person. The bad channels including loss of EEG signals or existence of lots of artifact signals were rejected. The artifact components include ocular artifacts, discontinuities, and muscle activity. No channel was discarded in this dataset. Second, the trial was rejected when the output force was far from the target force during the hold period (2.5–6 s) when the force was lower than 2N and higher than 6N (Figure 2). The average rejected trial was 10.82 ± 8.71 and 8.65 ± 7.80 (mean ± standard deviation) in the auditory feedback group and no auditory feedback group, respectively, for all participants (*N* = 17). There was no statistical difference between the auditory feedback group and no auditory feedback group in the number of the rejected trial using paired *t*-test (*t* = 1.41, *p* = 0.18). EEG was divided into the alpha band (8–12 Hz), the beta band (12–30 Hz), and the gamma band (30–45 Hz) in the following analysis.

### Data analysis

#### Average electromyography envelope

The average EMG envelope was obtained by the following steps: the raw EMG signal was high-pass filtered at 10 Hz, low-pass filtered at 400 Hz, then the filtered EMG was rectified and low-pass filtered at 5 Hz, and finally, the preprocessed data were averaged across all the people and trials in each condition (Chen et al., 2013).

#### Force variability

Force variability was computed by the standard deviation (SD) during the steady-hold period (Archer et al., 2018; Kang et al., 2019). It could measure the degree of disturbance of the output force and be defined as follow:


(1) $$forcevariability_{n}=SD\left(force_{n}\right)$$

*n* is the *j – th* data epoch.

#### Force accuracy

Force accuracy was computed by root mean square error (RMSE) during the steady-hold period (Fujii et al., 2016; Shafer et al., 2019; Lee and Kang, 2020). RMSE can evaluate the extent to which the applied force of participants deviated from the target force. It can be defined as follow:


(2) $$RMSE_{n}={\left(\sum_{i=1}^{N}\frac{{\left(s-f_{i}\right)}^{2}}{N}\right)}^{\frac{1}{2}}$$

*n* is the *j – th* data epoch, *s* is the target force, *f*_*i*_ is the *i – th* force sample, *N* is the number of data samples.

#### Power spectra estimation of electroencephalogram and electromyography

The previous study found that the alteration of power spectrum appeared in bilateral sensorimotor during unilateral hand movement at times and suggested that both contralateral and ipsilateral sensorimotor areas take part in unilateral hand movement (Crone et al., 1998; Lin et al., 2012; Ofori et al., 2015). Therefore, the power spectrum of bilateral sensorimotor was analyzed. This study used the left sensorimotor area (C3) and the right sensorimotor area (C4) as the research area of the power spectrum as previous research studies generally opted those brain regions for this kind of research work (Johnson and Shinohara, 2012; Abdalsalam et al., 2018).

Electroencephalogram and EMG signals were segmented into 1 s during the steady-hold period for the calculation of the power spectrum. To prevent the influence of volume conduction and reference electrode, the present study used the spherical splines method to obtain current source density (CSD) signals (Perrin et al., 1989). CSD signal was divided into the alpha band (8–12 Hz), the beta band (12–30 Hz), and the gamma band (30–45 Hz). EMG was rectified before calculating the power spectrum of EMG. The power spectrum of a signal was computed as follows (Challis and Kitney, 1991):


(3) $$P_{xx}\left(f\right)=\frac{1}{M}\sum_{i=1}^{M}X_{i}X_{i}^{{}^{*}}$$

Where *P*_*xx*_(*f*) is the power spectra density in channel *x* at a given frequency *f*. *X*_*i*_ is the Fourier transform of the segments *i* (*i* = 1,…, *M*) of channel *x*. * means complex conjugate. Power spectra density was computed employing the multitapers (Thomson, 1982; Mitra and Pesaran, 1999), and this method uses discrete prolate spheroidal sequences (DPSS) taper (Slepian and Pollak, 1961). Power spectra of a specific frequency band were normalized to the sum of the entire power of all frequencies (from 0 to 500 Hz) to obtain the power ratio.

#### Estimation of corticomuscular coherence

Electroencephalogram and EMG signals were segmented into 1 s during the hold period for the calculation of the CMC value. The average number of segments was 276.71 ± 34.85 and 285.41 ± 31.21 in the auditory feedback condition and no auditory feedback condition. There was no statistical difference between the auditory feedback group and the no auditory feedback group in the number of segments using paired *t*-test (*t* = –1.41, *p* = 0.18). The EMG signal was rectified, which was suitable for low-level force and was used in previous studies in the estimation of CMC (Kristeva et al., 2007; Johnson et al., 2011; Farina et al., 2013; Ward et al., 2013). CMC is assessed through the coherence between the specified CSD signal and rectified EMG signal (Nunez et al., 1997). Coherence is represented by the following definition:


(4) $$Coh_{xy}\left(f\right)=\frac{{\left|P_{xy}\left(f\right)\right|}^{2}}{P_{xx}\left(f\right)P_{yy}\left(f\right)}$$

Where *Coh*_*xy*_(*f*) is the coherence between CSD in channel *x* and rectified EMG in channel *y* at a given frequency *f*. *P*_*xx*_(*f*) and *P*_*yy*_(*f*) are the Fourier power spectra density for the CSD signal in channel *x* and the rectified EMG signal in channel *y* at frequency *f*, respectively. *P*_*xy*_ (*f*) is the Fourier cross-spectrum density for the CSD signal in channel *x* and the EMG signal in channel *y* at frequency *f*. The auto spectra and cross spectra were computed employing the multitapers, this method uses DPSS taper.

Due to the individual differences in CMC, the present study employed Z-score CMC before averaging all participants, the Z-score CMC was used as statistical input data (Chen et al., 2013; Li et al., 2020). The Z-score CMC was computed according to the following formula:


(5) $$Coh_{Z-score}\left(f\right)=\frac{Coh\left(f\right)-\mu_{Coh}\left(f\right)}{\sigma_{Coh}\left(f\right)}$$

Where μ_*Coh*_(*f*) and σ_*Coh*_(*f*) represent the mean value and standard deviation among all the channel EEG-EMG values for a given frequency bin.

Selection of electrode of interest: due to the difference between the head model and the wearing position of the electrode cap, the topography graph of the CMC of each participant is participants-specific in the sensorimotor cortex. Thus, before the statistical analysis, the present study defined FC3, C3, and CP3 as the left sensorimotor area. Because CMC appeared obviously and extensively in the beta band over the contralateral sensorimotor area during the unilateral hand steady-hold period according to the previous study (Kristeva et al., 2007; Witte et al., 2007). Therefore, the present study used the maximum sensor of beta band Z-scored CMC in the left sensorimotor area as the interesting channel for CMC analysis.

Topography graph: the topography graph was employed to determine the channel of interest. This procedure was conducted after averaging the Z-score CMC within each of the hold periods over the alpha, beta, and gamma frequency bands. Then, this study defined the maximum value of EEG–EMG electrodes across all the participants as a channel of interest for the time-frequency analysis and power spectrum analysis.

Time-frequency analysis: the time-frequency coherence (short-time Fourier transform coherence) is defined as follows (Zhan et al., 2006):


(6) $$Coh_{xy}\left(t,f\right)=\frac{{\left|P_{xy}\left(t,f\right)\right|}^{2}}{P_{xx}\left(t,f\right)P_{yy}\left(t,f\right)},t=1,\ldots ,N$$

Where *t* is time relative to the beginning of a trial. *N* is the time point of a trial. *Coh*_*xy*_(*t*, *f*) is the coherence between CSD in channel *x* and rectified EMG in the channel *y* at time *t* and frequency *f, P*_*xx*_(*t*, *f*) (*P*_*yy*_(*t*, *f*)) are Fourier power spectrum density in channel *x*(*y*) at time *t* and frequency *f*. *P*_*xy*_(*t*, *f*) is Fourier cross-spectral densit*y* for the CSD signal in channel *x* and the EMG signal in channel *y* at time *t* and frequency *f*. The time-frequency CMC is computed in all the trials and averaged between all the trials. In the time-frequency analysis of power spectrum density and cross-spectrum density in all the trials, a 1 s sliding time window (tw) with a sliding step of 0.1 s was applied. Due to the influence of boundary effects, the present study preprocessed data between 1 s before movement and 1.5 s after the movement, and computed spectra values from 0.5 s before movement to 0.5 s after movement. For each frequency bin, this study set the distance of frequency smoothing (fd) to plus-minus 8.5 Hz, these settings by Shannon number (Percival and Walden, 1993), that *tap* = 2*tw*fd-1, where a tap is the number of tapers and has to be higher than 0. The time-frequency analysis of auto spectra and CMC was computed in a typical participant. Time frequency analysis can show the dynamic changes of neural mechanisms with time and frequency (Morales and Bowers, 2022).

### Statistical analysis

For all statistical data, the first step is to carry out a normality test to determine its statistical method. This study used the “lillietest” MATLAB function (Kolmogorov-Smirnov test) to test the differences between the paired groups to conduct a normality test. All statistical variables are normally distributed or approximate normal distribution. Paired Student’s *t*-test (paired *t*-test) was used between the auditory feedback group and no auditory feedback group with 17 participants to test the effect of auditory feedback on the force amplitude, force RMSE, force SD, normalized power spectrum (alpha, beta, and gamma band over C3 and C4), and the amplitude of Z-score CMC (alpha, beta and gamma band) during the steady-hold period. In addition, results are expressed as the mean ± SEM (standard error of the mean). The significance level was set at *p* = 0.05.

## Results

### Motor performance

#### Average force amplitude

The average force amplitude of exerted force by the right hand was calculated during the steady-hold period in each task. The average force amplitude was 3.93 ± 0.05 and 3.77 ± 0.08 in the auditory feedback group and no auditory feedback group, respectively. The paired *t*-test revealed that the average force amplitude is higher in the auditory feedback condition compared with the no auditory feedback condition (*t* = 4.07, *p* < 0.001).

#### Average force envelope and average electromyography envelope

**FIGURE 3:**
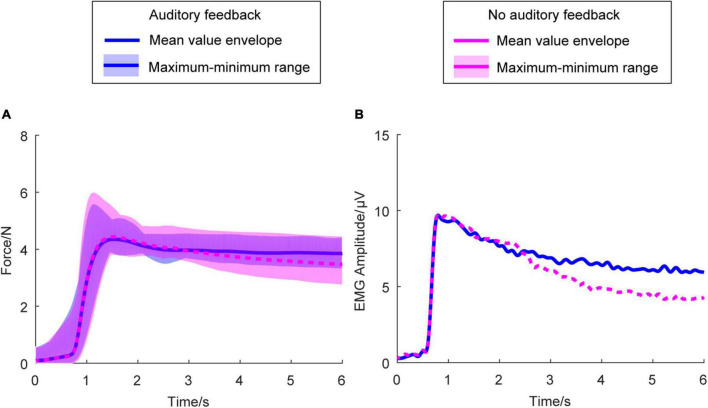
Average force envelope and average EMG amplitude in all the participants (*N* = 17) and two conditions. In **(A)**, the horizontal axis means the time, the vertical axis means the force amplitude. In **(B)**, the horizontal axis means the time, the vertical axis means the EMG amplitude. The transparent area means the maximum and minimum range of the mean force exerted force of each participant across all the participants at every sampling point. The blue solid line means the mean value envelope across all the participants in the auditory feedback condition, the magenta dashed line means the mean value envelope across all the participants in the no auditory feedback condition. EMG, electromyography.

Figure 3A shows the average right-hand force envelope for all the participants. The blue line means the auditory feedback condition, the magenta line means no auditory feedback condition. The envelope of the exerted force means the maximum and minimum of the mean force of each participant across all the participants at every sampling point. Figure 3B is the average envelope of the right-hand EMG. Figure 3 indicates the kinematic behavior of all the participants within a limited range and meets the exerted force standard.

#### Average force standard deviation and average force root mean square error

**FIGURE 4:**
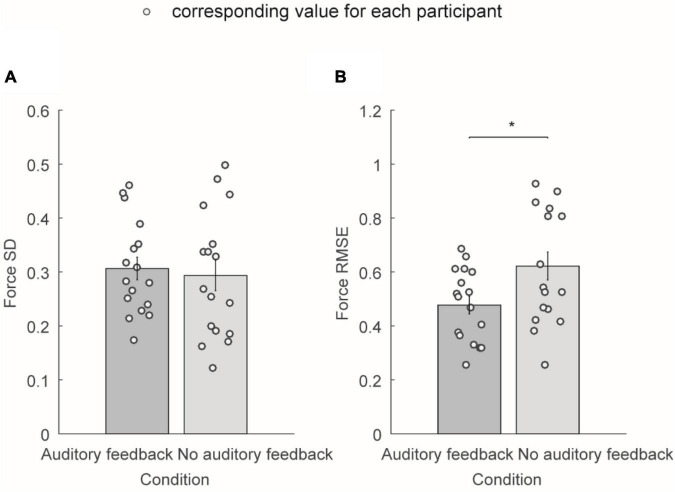
The average force SD and average force RMSE of all the participants (*N* = 17) in auditory feedback and no auditory feedback conditions. **(A)** The average force SD in the auditory feedback group and no auditory feedback group, the horizontal axis means the condition type, and the vertical axis means the average force SD. **(B)** The average force RMSE in the auditory feedback group and no auditory feedback group, the horizontal axis means the condition type, and the vertical axis means the average force RMSE. The empty circles mean the corresponding value for each participant. The data were presented as mean ± SEM. *p < 0.05. SEM, standard error of the mean; SD, standard deviation; RMSE, root mean square error.

Figure 4 shows the average force SD and average force RMSE of the exerted force in the right-hand during the steady-hold period. Figure 4A shows the average force SD in the auditory feedback group and no auditory feedback group, the average force SD was 0.31 ± 0.02 in the auditory feedback group, and 0.29 ± 0.03 in the no auditory feedback group. There is no significant difference in the average force SD between the auditory feedback group and the no auditory feedback group (*t* = 0.60, *p* = 0.56). Figure 3B shows the average force RMSE in the auditory feedback group and no auditory feedback group, the average force RMSE was 0.48 ± 0.03 in the auditory feedback group, 0.62 ± 0.05 in the no auditory feedback group. The paired *t*-test revealed that the auditory feedback group was lower than the auditory feedback group in average force RMSE (*t* = –4.85, *p* < 0.001).

### Power spectrum and corticomuscular coherence

#### Time frequency plots of the power spectrum and corticomuscular coherence spectrum

**FIGURE 5:**
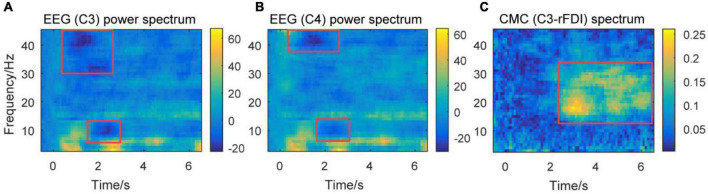
Time-frequency plots of percent change of power spectra of EEG signals, and CMC spectrum from a representative participant. Time frequency plots of percent change of EEG power spectrum over channels C3 **(A)** and C4 **(B)** from a representative participant. Time frequency plots of CMC spectrum between channel C3 and EMG in rFDI muscle **(C)** from a representative participant. The horizontal axis means the time, the vertical axis means frequency. EEG, Electroencephalogram; EMG, electromyography; rFDI, right first dorsal interosseous; CMC, corticomuscular coherence.

Figure 5 demonstrates the corresponding percent change of power spectra of EEG signals (recorded from channels C3 and C4), and CMC (C3-rFDI) spectrum respectively from a representative participant. Time-frequency plots can show how the percent change of EEG power spectrum and CMC spectrum changed over frequency and time. The percent change of time-frequency power was computed as follows:


(7) $$percentchange\_timefrequencypower_{tf}=\frac{power_{tf}-meanbaseline_{f}}{meanbaseline_{f}}\times 100$$

The baseline for time frequency power was set at –0.5 to 0 s. Figures 5A,B show the power of the theta band increasing at the beginning of the movement and steady-hold period. This result accords that theta band is associated with movement initiation and execution (Ketenci and Kayikcioglu, 2019). The power of alpha and gamma bands decreases at the beginning of the movement. The decrease of the gamma band prior to the decrease of the alpha band at the beginning of a movement. This result accords that the gamma band is associated with movement adjustment (Mehrkanoon et al., 2014). The decrease in alpha band power is associated with the excitability of the sensorimotor area (Pfurtscheller and da Silva, 1999). Figure 5C shows that the CMC spectrum (C3-rFDI) is significant in 12–35 Hz, mainly distributed in alpha, beta, and gamma bands during the steady-hold period. Thus, the present study focuses on the alpha, beta, and gamma bands in the subsequent analysis.

#### Power spectra

**FIGURE 6:**
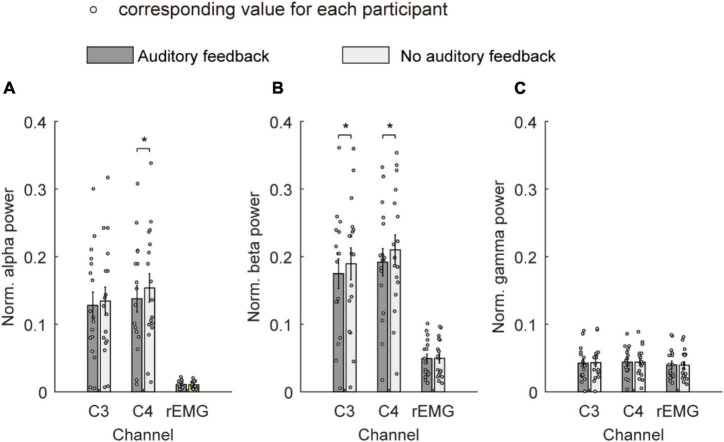
The average normalized power spectrum of all the participants (*N* = 17) in the alpha band **(A)**, the beta band **(B)**, and the gamma band **(C)** over channels C3, C4, and rEMG in auditory feedback and no auditory feedback conditions, the horizontal axis means the channel label, the vertical axis means the average normalized power spectrum. The empty circles mean the corresponding value for each participant. The data were presented as mean ± SEM. *p < 0.05. SEM, standard error of mean; rEMG, right electromyography.

Figure 6 shows the average normalized power spectrum of the alpha band, beta band, and gamma band over channels C3, C4, and rEMG in the auditory feedback group and no auditory feedback group. The average normalized power spectrum of the alpha band was 0.13 ± 0.02 and 0.13 ± 0.02 over channel C3 in the auditory feedback group and no auditory feedback group, respectively. The average normalized power spectrum of the alpha-band was 0.14 ± 0.02 and 0.15 ± 0.02 over channel C4 in the auditory feedback group and no auditory feedback group, respectively. The average normalized power spectrum of the alpha-band was 0.01 ± 0.001 and 0.01 ± 0.001 over channel rEMG in the auditory feedback group and no auditory feedback group, respectively. Paired *t*-test revealed that auditory feedback group was lower than no auditory feedback group over channel C4 (*t* = –4.15; *p* < 0.001), but not channel C3 (*t* = –1.61, *p* = 0.13) and rEMG (*t* = 1.83, *p* = 0.09) in the average normalized power spectrum of the alpha band.

The average normalized power spectrum of the beta band was 0.17 ± 0.02 and 0.19 ± 0.02 over channel C3 in auditory feedback and no auditory feedback. The average normalized power spectrum of the beta band was 0.19 ± 0.02 and 0.21 ± 0.02 over channel C4 in auditory feedback and no auditory feedback. The average normalized power spectrum of the beta band was 0.05 ± 0.01 and 0.05 ± 0.01 over channel rEMG in auditory feedback and no auditory feedback. Paired *t*-test revealed that auditory feedback group was lower than no auditory feedback group over channel C3 (*t* = –2.70, *p* = 0.02) and C4 (*t* = –2.87, *p* = 0.01) but not channel rEMG (*t* = –0.12, *p* = 0.91) in normalized power spectrum of beta band.

The average normalized power spectrum of the gamma band was 0.04 ± 0.01 and 0.04 ± 0.01 over channel C3 in the auditory feedback condition and no auditory feedback condition. The average normalized power spectrum of the gamma band was 0.04 ± 0.01 and 0.04 ± 0.01 over channel C4 in auditory feedback and no auditory feedback. The average normalized power spectrum of the gamma band was 0.04 ± 0.01 and 0.04 ± 0.01 over channel rEMG in auditory feedback and no auditory feedback. Paired *t*-test revealed that normalized power spectrum of the gamma band was not influenced by task type over channel C3 (*t* = –0.52, *p* = 0.61) and C4 (*t* = 0.11, *p* = 0.91) and rEMG (*t* = 0.68, *p* = 0.50).

#### Corticomuscular coherence

**FIGURE 7:**
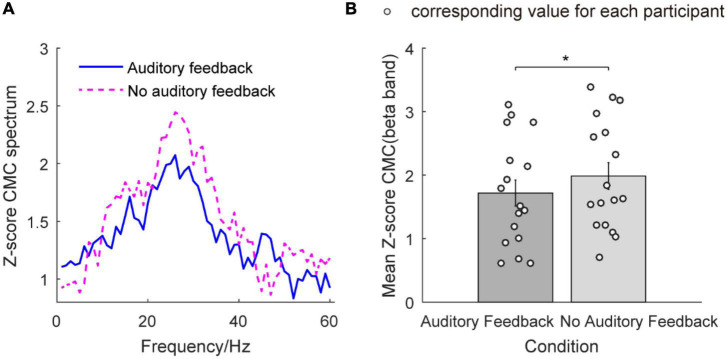
The Z-score CMC from 1 to 60 Hz and mean Z-score CMC of the beta band in the auditory feedback condition and no auditory feedback condition in all the participants (*N* = 17). **(A)** The Z-score CMC across 1–60 Hz in each condition type, the horizontal axis means frequency, and the vertical axis means the value of the Z-score CMC spectrum. The blue solid line means auditory feedback group, the magenta dashed line means no auditory feedback group. **(B)** The mean Z-score CMC of the beta band in each task, the horizontal axis means condition type, and the vertical axis means the value of mean Z-score CMC of the beta band. The empty circles mean the corresponding value for each participant. The data were presented as mean ± SEM. *p < 0.05. SEM, means standard error of mean; CMC, corticomuscular coherence.

Figure 7 illustrates the Z-score CMC from 1 to 60 Hz and the mean Z-score CMC of the beta band in the auditory feedback condition and no auditory feedback condition. In Figure 7A, the peaks of Z-score CMC appear in the beta band (26 Hz) in each task type. Figure 7B illustrates the mean Z-score CMC of the beta band in two condition types. The average Z-score CMC of the beta band was 1.72 ± 0.21 and 1.99 ± 0.21 in the auditory feedback condition and no auditory feedback condition. Statistical analysis revealed a significantly increased Z-score CMC of the beta band in the no auditory feedback condition compared with the auditory feedback condition during the steady-hold period by the right hand (*t* = –2.22, *p* = 0.04). No significant difference was found in Z-score CMC of the alpha band (*t* = –0.64, *p* = 0.53) and gamma band (*t* = –1.33, *p* = 0.20) (*p* > 0.05) between no auditory feedback condition and auditory feedback condition during the steady-hold period by the right hand.

#### The spatial topography map of corticomuscular coherence in alpha, beta, and gamma bands

**FIGURE 8:**
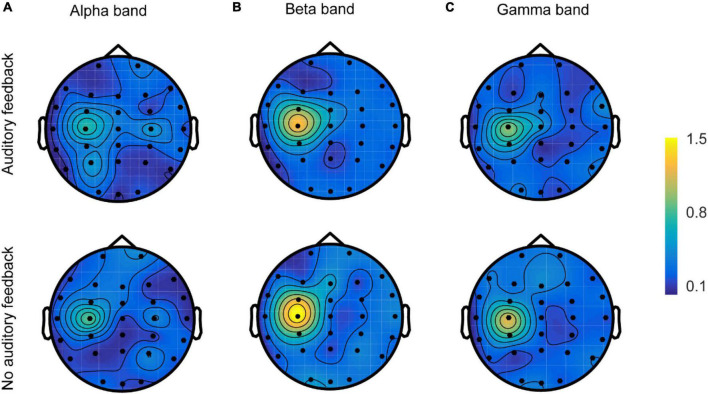
The spatial topologies of CMC of all the participants (*N* = 17) in the alpha band **(A)**, the beta band **(B)**, and the gamma band **(C)** in auditory feedback condition and no auditory feedback condition. CMC, corticomuscular coherence.

The spatial topologies of CMC for the alpha, beta, and gamma bands during each task are shown in Figure 8. All six topologies revealed maximal CMC in the channel over the contralateral sensorimotor area (C3) in alpha, beta, and gamma bands, but there were obvious differences in their distributions. Beta band and gamma band CMC showed a single maximum over channel C3, whereas the gamma band was generally weaker. Alpha band CMC revealed a more disturbing pattern with multiple maxima and sub-maxima. In addition to the maximum channel C3, two sub-maxima were observed over channels P3 and C4 in the auditory feedback condition, and over channels C4 and P4 in the no auditory feedback condition. In each frequency, the maximum of the topology of the auditory feedback condition was generally weaker compared with the maximum of the topology of the no auditory feedback condition.

## Discussion

The present study researched how regulating auditory feedback regarded behavior, brain activation level, and neuromuscular activity during the thumb and index finger pinching motion of the right hand. We found three main results. First, the auditory feedback condition resulted in decreased movement error when compared with no auditory feedback condition during the steady-hold period. Second, the auditory feedback condition resulted in decreased mean normalized power spectrum in the alpha band over channel C4 and decreased mean normalized power spectrum in the beta band over channels C3 and C4 when compared with no auditory feedback condition. Third, we also found decreased mean Z-score CMC of the beta band between rEMG and contralateral sensorimotor area during the auditory feedback condition compared to the no auditory feedback condition in the beta band. Besides, there was the maximum CMC spectrum over C3 in alpha, beta, and gamma bands. These novel findings indicated that brain activation activity and a decrease in neuromuscular activity may coordinate to facilitate precise hand motion.

The auditory feedback group showed no significant difference in force variability compared with the no auditory feedback group during the steady-hold period in hand pinching movement. Force variability measures the degree of disturbance of the output force. This non-significant difference may come from methodological issues. Methodological issues are the range of retained output force, we limit the range of retained output force to a narrow range. This procedure can preserve the force output of the target force range and delete the data that is too far away from the target force. Therefore, the range of retained output force is an important parameter for the analysis of auditory feedback task in force variability.

The mean force is lower in the auditory feedback condition compared with the no auditory feedback condition. The result shows that the lack of feedback information decreases the amplitude of exerted force. Previous research studies found the exerted force decreases in low visual gain conditions compared with high visual gain conditions (Hong et al., 2008; Coombes et al., 2011). Besides, the mean force has a downward trend in the no auditory feedback condition. This phenomenon indicates that exerted force may be influenced by time in no auditory feedback condition. Without the provision of auditory feedback information, the exerted force is more likely to deviate from the target force.

The auditory feedback group showed a significant decrease in force error compared with the no auditory feedback group during the steady-hold period in hand pinching movement. Force RMSE assesses the accurate degree to which exerted force of the muscle conducts a movement task that deviates from the target force (Fujii et al., 2016). The results showed that movement was more accurate in the auditory feedback condition compared with the no auditory feedback condition. This result indicated auditory feedback provided essential and related information for guiding accurate motor avoiding deviating from the target force. A similar result was found in the previous study (Rosati et al., 2012; Gao et al., 2018). However, the prior studies introduced irrelevant variables such as visual information, which may influence the effect of auditory feedback on force accuracy. This study avoided unnecessary factors and focused on auditory modality to explore this question.

The auditory feedback group showed decreased normalized power spectrum compared with the no auditory feedback group over channel C4 in the alpha band during unilateral right-hand maintenance movement. Alpha oscillations can regulate cortical excitability, previous studies found that the reduction of alpha oscillations, named alpha suppression, indicated cortex activation, therefore, enhancing sensory responses to stimuli (Pfurtscheller et al., 1996; Haegens et al., 2011; Klimesch, 2012). This result showed that the brain was more active in the auditory feedback condition compared with the no auditory feedback condition during unilateral hand maintenance movement. When the contraction motor task receives auditory feedback. The ipsilateral sensorimotor area has a significant difference. A similar phenomenon occurred in the other experiments. Lin et al. (2012) found alpha band power suppression of the ipsilateral motor cortex with haptic feedback compared with no haptic feedback in the visual tracking task. Shibuya et al. (2014) utilized near-infrared spectroscopy in the bilateral cortex, the result showed that the contralateral sensorimotor cortex had significant differences only in weak contraction vs. strong contraction and moderate contraction vs. strong contraction, however, the ipsilateral sensorimotor cortex showed the significant difference existed between any two task conditions.

The auditory feedback group showed decreased normalized power spectrum compared with the no auditory feedback group over channels C3 and C4 in the beta band. An accumulating body of evidence emphasizes that the beta band was associated with maintaining the sensorimotor function (Kristeva et al., 2007; Mehrkanoon et al., 2014). Gilbertson et al. (2005) investigated that the increase of the beta band is related to impairment in motor action. Kuhn et al. (2008) used high-frequency stimulation to stimulate the subthalamic nucleus in patients with Parkinson’s disease, resulting in beta oscillations suppression in the motor cortex and better performance. Our result supported this view that the beta band plays an important part in maintaining the sensorimotor system state, besides, the current result extended the view that the beta band was an efficient index to embody different sensorimotor maintenance states in auditory feedback and no auditory feedback during unilateral accurate hand motor. This mechanism of decreased power spectrum was similar to event-related desynchronization. Many studies have found that beta band event-related desynchronization occurs on the bilateral sensorimotor area during unilateral hand maintenance movement, which is very robust (Crone et al., 1998; Pfurtscheller and da Silva, 1999). Taken together, these results suggested that auditory feedback causes the more active sensorimotor cortex compared with no auditory feedback in the alpha and beta band during unilateral hand maintenance movement.

Corticomuscular coherence of the beta-band decreased in the auditory feedback condition compared with no auditory feedback condition in the steady-hold period during a right-hand pinching task. CMC is a synchronization indicator between the sensorimotor area and the effector’s muscle, which is deed to be the key to practical motor control, it is generally assumed that CMC is embody efferent neurotransmission and can assess the strength of the cortical-muscle pathway (Mima and Hallett, 1999). In healthy participants, the beta band is regarded as an indicator to evaluate the functional pattern of sensory and motor systems (Kristeva-Feige et al., 2002; Engel and Fries, 2010; de Vries et al., 2016; Chen et al., 2018; Kasuga et al., 2018; Watanabe et al., 2020). The present study found CMC of the beta band decreased in the auditory feedback condition compared with the no auditory feedback condition. Force level and attention load affect the amplitude of CMC (Kristeva-Feige et al., 2002; Witte et al., 2007; Chakarov et al., 2009). Previous studies found the amplitude of CMC is higher at higher force during low level force (Witte et al., 2007; Chakarov et al., 2009). Our result shows that the mean force is lower in the no auditory feedback condition compared with the auditory feedback condition significantly. However, the difference between the two conditions is very small and lower than the precision of exerted force. Therefore, the force level is not the main factor affecting the amplitude of CMC in our study. Attention is another important factor affecting the amplitude of CMC. The result showed that the CMC of the beta-band decreased when the attention load is increasing between the motion task and auditory feedback information simultaneously supported. The decrease in CMC may account for the increasing task load and lead to divided attention. The auditory feedback condition enhances the feedforward and feedback loops, then need to deal with a more complex task load than the no auditory feedback condition. Previous studies found similar results. Kristeva-Feige et al. (2002) found that the CMC of beta-band decreased when participants conducted highly precise visual feedback motor and mental arithmetic tasks compared with highly precise motor only. Johnson et al. (2011) found CMC of the beta-band decrease in bilateral motor and motor cognitive tasks compared with a unilateral motor task, he speculated that the attenuation of CMC was due to divided attention. Recently, Li et al. (2020) found a significant decreased mean Z-score CMC of the beta band between effector hand and contralateral brain area in the tactile stimulation compared with no tactile stimulation, the author also conjectured that the attenuation of CMC due to the divided attention. Besides, CMC of the beta band serves as the vital marker for coordinate control of motor neurons, and is related to the lowest computational energy. The cortico-muscular connection may carry out in decreased pattern when the attentive resources are toward the sensorimotor sequence (Kristeva-Feige et al., 2002). The current result is in favor of the viewpoint that CMC of the beta-band is related to attention toward sensorimotor information and plays a vital part in auditory-motor control. This study also found that the CMC of the beta band was strongest in the contralateral sensorimotor area. The sensorimotor area may play an important part in the motor and auditory-motor mapping that requires the output of accurate force with auditory and motion messages (Chen et al., 2013).

Regarding the effects of auditory feedback on dominant hand force pinch control, we need to consider some limitations. First, given that only healthy young participants participated in this study, we are not certain whether our findings could be generalized to other populations with larger motor output variability such as children, elderly adults, and neuromuscular sufferers. Second, our findings are restricted to low contraction levels. We are not certain whether our findings could be generalized to other force levels or another auditory feedback mode. Thus, future studies should be carried out on how different force contraction levels and specific parameters of auditory feedback affect neurophysiology and behavioral output. Third, the present study focuses on the auditory feedback factor, the impact of gender on the results would be investigated in the future study.

The results of the current study have potential applications for sports training (e.g., precise shooting) and hand function rehabilitation. For healthy athletes, using auditory feedback technology in the process of training can improve shooting performance. Besides, a potential application of auditory feedback is associated with the hand function rehabilitation of patients with stroke. In the rehabilitation of hand function after stroke, auditory feedback can be used in occupational therapy such as holding a pen (two fingers pinch), pinching a spoon (three fingers pinch), and holding a cup (grasp). Auditory feedback can be a potential technology to improve hand flexibility and control ability. Auditory feedback technology (such as virtual reality rehabilitation training, and music support therapy) has been applied in the hand function rehabilitation of patients with stroke. This study researched functional connectivity circuits during auditory feedback tasks and provided the neural mechanism for the change of brain plasticity in exercise training or motor rehabilitation of patients with stroke. The findings of the present study for CMC analysis would be explored further in the follow-up studies for patients with stroke.

The present study investigated the effects of auditory feedback during the fine thumb and index finger pinching tasks. The results from the study indicated that auditory feedback contributed to increased movement accuracy, decreased alpha power spectrum in the ipsilateral sensorimotor area and beta power spectrum in the bilateral sensorimotor area, and decreased beta band CMC compare with no auditory feedback. The present study indicates that auditory feedback caused the improvement of movement accuracy accompanied by suppression of oscillation and a decrease of corticospinal connectivity during precise hand movement. This regulation mechanism allows the brain to perform better with limited energy through intelligent management. When patients have a limited capacity for motor control (e.g., in the initial stage of rehabilitation or patients with poor motor control), auditory feedback may be a proper auxiliary rehabilitation technique to assist patients to achieve motor control with limited energy. This study supports a new perspective on rehabilitation strategy. Future studies should examine patients with movement disorders to test this mechanism. The behavioral results and neurophysiology mechanism presented here have a significant impact on our understanding of the role of auditory feedback in brain activity and the corticospinal pathway that support this control. Meanwhile, these findings have a significant impact on auditory feedback training in rehabilitation settings such as virtual reality rehabilitation equipment, sports equipment, and brain-computer interface.

## Data availability statement

The datasets generated and/or analyzed during the current study are not publicly available due to data privacy but they are available from the corresponding author on reasonable request. Requests to access the datasets should be directed to the corresponding author, JW.

## Ethics statement

The studies involving human participants were reviewed and approved by the Ethics Committee of Medical College of Xi’an Jiaotong University. The patients/participants provided their written informed consent to participate in this study.

## Author contributions

JG designed, performed the experiment, analyzed the data, prepared the figures, drafted the manuscript, and modified the grammar. JG and TL interpreted the results of the experiment. JG, TL, and JW edited, revised the manuscript, and approved the final version of the manuscript. All authors contributed to the article and approved the submitted version.

## Abbreviations

CMC: corticomuscular coherence
EEG: electroencephalogram
EMG: electromyography
rFDI: right first dorsal interosseous
CSD: current source density
SD: standard deviation
RMSE: root mean square error
DPSS: discrete prolate spheroidal sequences
rEMG: right electromyography.
